# Changing Trends in Prevalence and Antibiotics Resistance of Uropathogens in Patients Attending the Gondar University Hospital, Northwest Ethiopia

**DOI:** 10.1155/2014/629424

**Published:** 2014-03-10

**Authors:** Moges Tiruneh, Sisay Yifru, Mucheye Gizachew, Kassie Molla, Yeshambel Belyhun, Feleke Moges, Mengistu Endris

**Affiliations:** University of Gondar, P.O. Box 196, Gondar, Ethiopia

## Abstract

*Background*. In most hospitals of developing countries, urinary tract infections are treated empirically because of lack of culture facilities. This leads to emergence of multiresistant uropathogens. Culturing and drug susceptibility testing are essential to guide therapy. *Objectives*. To assess changing prevalence and resistance pattern of uropathogens to commonly used antibiotics in a two-year study period. * Methods*. Urine specimens were collected and cultured. Uropathogens were identified by standard methods and tested for antibiotics resistance. Data were analyzed using SPSS version 16 statistical sofware. * P* value < 0.05 was considered statistically significant. * Results*. The commonest isolates in both the previous and present studies were * E. coli*, *Klebsiella*, CoNS, * S. aureus*, * Proteus*, and * Citrobacter* species. Previous isolates of Enterobacteriaceae were 100% sensitive to ciprofloxacin, whereas present isolates developed 31% to 60% resistance to it. Previous isolates were less resistant to gentamycin than the present ones. Multiresistance isolates were predominant in present study than previous ones. *Conclusion*. * E. coli* was predominant in the two study periods. Present isolates were more resistant than previous ones. Some previous isolates were 100% sensitive to ciprofloxacin, whereas present isolates were increasingly resistant. Ciprofloxacin and gentamicin have been recommended for empiric treatment of urinary tract infections.

## 1. Background

Urinary tract infection is one of the commonest bacterial infections encountered in daily clinical practice [1]. It has been estimated that worldwide about 150 million people suffer from asymptomatic and symptomatic UTIs each year [2]. In most parts of the sub-Saharan Africa, as well as in other developing parts of the world, UTI is among the most common health problems occurring both in the community and hospitalized patients [3]. Since the last two to three decades, just as many community and hospital acquired bacterial infections, UTIs due to multidrug resistant uropathogens have caused a growing concern worldwide [1, 4–6]. Investigators [1, 7, 8] explained that the drug resistance problem in Africa stems from factors like indiscriminate use of antibiotics, inappropriate advertisement, and erratic prescription by unqualified drug sellers.

Since the previous two decades, the problem of UTIs due to uropathogens resistant to the commonly used antibiotics was reported by many authors in Ethiopia in general and in Gondar region in particular [1, 9–12]. Consequently, the prevalence of urinary tract pathogens and their resistance to the different antibiotics may have changed over the years in the study area. Hence, studies are needed as a guide in the community and hospital health care settings. The objectives of this study therefore were to assess the changing prevalence and resistance patterns of the uropathogens to commonly used antibiotics in a two-year study period.

## 2. Materials and Methods

### 2.1. Study Design and Area

This hospital based cross-sectional study was conducted at the Gondar University hospital from September 1, 2011, to June 30, 2012. Gondar University hospital is found in Gondar town, located 727 km from Addis Ababa to the Northwest Ethiopia. Gondar town has a population of about 207,000 [13].

### 2.2. Sample Collection and Processing

Urine samples were collected by a clean-catch midstream, catheterization, or use of urine bags in a sterile container from each study participant whom the clinicians suspected for UTI and who has not received antibiotic therapy during the previous 14 days. Isolation of uropathogens was done by a surface streak procedure on Cysteine Lactose Electrolyte Deficient (CLED) (Oxoid, LTD, UK) medium using a 0.001 mL calibrated inoculating wire loop. After 18–24 hours of incubation at 37°C aerobically, colonies were counted and multiplied by 1000 which resulted in ≥10^5^ colony forming unit (CFU)/mL of urine sample. Subculture of the colonies was done on blood agar (Oxoid, LTD, UK) and MacConkey agar (Oxoid, LTD, UK) to characterize the isolate.

Bacterial identification was then done by standardized biochemical tests, namely, indole production, lactose fermentation, hydrolysis of urea, citrate utilization, lysine decarobxylation, motility test, oxidase test for gram negative bacteria and for gram positive bacteria, mannitol fermentation, and catalase and coagulase tests [14].

### 2.3. Susceptibility Testing

Antibiotic susceptibility was tested for all the isolates by the disc diffusion techniques according to Bauer et al. [15] and Clinical Laboratory Standard Institute (CLSI) guidelines [16]. The pure culture colony suspension of the isolate was made using sterile physiological saline and adjusted to 0.5 McFarland standards. Muller Hinton agar plate was swabbed with the suspension using sterile cotton swap and the antibiotic discs were placed over the agar and left for 30 minutes for diffusion of the antibiotics in the disc. The plates were inverted upside down and incubated at 37°C for 18 to 24 hours. The zones of inhibition were then read as resistant and sensitive using calibrated ruler and compared with the standard chart [15]. Intermediate results were few in number and therefore were considered as resistant for convenience. Antibiotics agents employed for susceptibility testing were ampicillin (10 *μ*g), amoxicillin (30 *μ*g), co-trimoxazole (25 *μ*g), gentamicin (10 *μ*g), ciprofloxacin (5 *μ*g), penicillin (10 IU), and erythromycin (15 *μ*g) (Oxoid, Ltd, UK).

This study was done using the same method conducted by Moges et al., 2002 [1], 10 years back in the same study area, and the present results were compared with the previous report [1].

### 2.4. Quality Control

Each batch of the culture media used was tested for sterility. Standard control strains of* E. coli* ATCC 25922 and* S. aureus* ATCC 25923 were used during culturing and antibiotics susceptibility testing as a control throughout the study.

### 2.5. Data Analysis

Data were checked for completeness, cleaned manually, entered, and analyzed using SPSS version 16 statistical software. The chi-square test (*χ* ^2^) was used to measure the association and a* P* value less than 0.05 was considered statistically significant.

### 2.6. Ethical Clearance

Ethical clearance was obtained from the research and publication office of the University of Gondar, College of Medicine and Health Sciences. The data were collected after a written informed consent was sought from each study participant.

## 3. Results

Of the total 538 consecutive urine samples cultured, 284 (52.8%) gave significant bacteriuria. The majority (58.8%) of the positive cases were females while the remaining (41.2%) were males. The age of the study participants ranged from 1 year to 50^+^ years with the median age of 24. The frequency of positive urine cultures (28.2%) was high in the age group of 20–29 years followed by the age group of 50^+^ years (Table 1).

As it is seen from Table 2, the most common isolate of the uropathogens was* E. coli*, (42.3%), followed by* Klebsiella* spp., (14.4%), CoNS, (12.3%),* S. aureus* (9.2%),* Proteus* spp., and* Enterobacter* spp. (5.6%).* Klebsiella* spp. showed the highest rate of resistance to amoxicillin (97.7%), ampicillin (95.1%), tetracycline and cotrimoxazole (82.9%), and chloramphenicol (78%) compared to* E. coli *to the same antibiotics.* Citrobacter*,* Enterobacter*,  and* Proteus* species revealed higher rate of resistance patterns to tetracycline, ampicillin, amoxicillin, cotrimoxazole, and chloramphenicol.* S. aureus* and CoNS also demonstrate higher resistance rates to tetracycline, cotrimoxazole, amoxicillin, ampicillin, and penicillin. All gram negative uropathogens were resistant to 25–60% ciprofloxacin, while 53.8%* S. aureus* and 54.3% CoNS were resistant to this antibiotic.

Referring to Table 3, of the 282 total urinary bacterial isolates tested for antibiotics resistance patterns, 86.5% have shown multidrug resistance (resistant to ≥2 antibiotics). The rest,* Enterobacter*,* Proteus*,* Streptococcus*,* Providencia*, and* Pseudomonas* spp., were resistant to five antibiotics tested, whereas* Citrobacter* spp. were resistant to four antibiotics.

Most of the uropathogens' isolates compared in the two study periods (2002 and 2012) showed no statistically significant differences in the isolation rates, except* S. aureus* which revealed a statistically significant decrease (18% to 9.2%) (*P* = 0.002) and* Enterobacter* spp. which revealed a significant increase (1.7% to 5.6%) (*P* < 0.001) in the current study (Table 4).

Table 5 shows that the resistance rate (34.2%) of* E. coli *for gentamicin in the present study was significantly higher than the rate (14.1%) of ten years back (*P* = 0.04). Similarly, the resistance rate (38.4%) of* S. aureus* isolates for ciprofloxacin in our study is significantly higher than the rate (6.5%) of the previous study (*P* = 0.03). The uropathogens (*E. coli, Klebsiella* spp., CoNS,* Citrobacter* spp., and* Proteus* spp.) in the previous study were found to be 100% sensitive to ciprofloxacin except the few isolates of* S. aureus* 2 (6.5%).

Table 6 shows that* E. coli* was more resistant to the tested antibiotics in our study than the previous ones.* Klebisella* spp. was resistant to seven antibiotics in this study than the previous ones which were resistant to five antibiotics. Previous isolates of* Proteus* spp. were less resistant to three and four antibiotics than in the present study.* Citrobacter* spp. of the present study were resistant to six antibiotics, whereas they were resistant to five in the previous study. CoNS and* S. aureus* each showed resistance to seven antibiotics in both study periods.

## 4. Discussion

In this study, the overall rate of isolation of the uropathogens was significantly higher than the previous rate (41%) reported from the same study area [1] but lower than the rate reported in Nepal (71.7%) [17].This disparity of rates may be attributed to the differences in the study samples and improper collection and processing of specimens. In our study, women have higher rate of UTI than men (Table 1), because in females the urethra has been known to be shorter and closer to the anus [18]. Other investigators have also reported similar findings to ours [5, 19]. The high prevalence of UTI in age groups of 20–29 and more than 50 years may be related to sexually active age group and older study participants whose immune system may be impaired, respectively.

The distribution of the uropathogens isolated in our study was almost similar to the results reported previously [1] except with* S. aureus*. The most commonly isolated uropathogen in the present study was* E. coli* and it is consistent with the previous study done in the same study area [1]. However, the frequency of* E. coli* in urine samples varies in different studies [20, 21]: these may be due to the large variation of different species of bacteria in the study and variations in specimen collection and processing.

Antibiotic resistance is a major clinical problem in treating infections caused by different bacterial pathogens. Resistance to antibiotics has increased over the years. In our present study, a higher proportion of isolates of members of Enterobacteriaceae was in average resistant to ampicillin (84%), tetracycline (83%), amoxicillin (80%), cotrimoxazole (77%), chloramphenicol (69%), gentamicin (50%), and ciprofloxacin (45%), whereas the resistance patterns of these isolates, a decade ago [1], were ampicillin (81%), tetracycline (78%), cotrimoxazole (67%), gentamicin (36%), and ciprofloxacin (0%). As has been clarified here, the most significant change among uropathogens in 10 years period has been the significant increase of resistance to gentamicin, and the emergence of resistance to ciprofloxacin from zero in the study conducted 10 years ago, to 45% in our case.

In comparing the two periods of study (2002 versus 2012) (Table 5), the resistance rate of* E. coli *(44.2%) for gentamicin in the present study was significantly higher than the rate (14.1%) of the previous study (*P* = 0.04). This finding is higher than studies from France (1.6%) [22] and Iran (30.1%) [23]. This decreasing trend in activity of gentamicin against major uropathogens such as* E. coli* raises a great concern regarding the empirical treatment of urinary tract infections.

Similarly, the resistance rate of* S. aureus* isolates for ciprofloxacin in our study is significantly higher than the rate (6.5%) of the previous study (*P* = 0.03) [1] which is supported by a report from teaching hospital in Saudi Arabia where there was high ciprofloxacin resistance in gram positive cocci, particularly* S. aureus *[24]. Hence, the overall trend of the present study indicates the emergence of increasing number of different multidrug resistant isolates of uropathogens than those in the previous study. Increased resistant patterns of the isolates for ciprofloxacin may be explained by the fact that the widespread use of the drug in the area might have favored the resistant isolates due to selective pressure.

Multiple drug resistance clinical isolates are increasing every time and some of the drugs are approaching to be no more essential for the treatment of UTI patients [17, 24]. The overall trends of the present study indicated that there are increasing multidrug resistant spp. in the present study (Table 3) compared to the previous isolates [1]. These studies urge a need for a large scale monitoring of drug resistance problems in different parts of the country and evaluate susceptibility patterns of the isolates. This would have a paramount importance in using empiric treatment which would be safe, effective, and economical for the patient.

## 5. Conclusion

This study has showed more resistant* E. coli *and* Klebsiella *spp. and* S. aureus* and CoNS to seven antibiotics tested over a ten-year period. The resistance rates of* E. coli *for gentamicin and* S. aureus *for ciprofloxacin in the present study were also higher than the rates of a decade ago. The uropathogens (*E. coli, Klebsiella* spp., CoNS,* Citrobacter* spp., and* Proteus* spp.) ten years back were found to be all sensitive to ciprofloxacin except the few isolates of* S. aureus*. Gentamicin and ciprofloxacin have been recommended for empirical treatment of urinary tract infections in areas where diagnostic bacteriologic services are not available.

## Figures and Tables

**Table 1:** Frequency of positive urine cultures for bacteria by age and sex in UTI suspected patients attending Gondar University Hospital, Northwest Ethiopia, 2012.

Variable	Frequency of positive isolates *N* (%)	*χ* ^2^	P value
Sex		45.8	0.007
Male	117 (41.2)		
Female	167 (58.8)		
Total	**284 (100.0)**		
Age group in years		2.1	0.32
<5	29 (10.2)		
5–9	13 (4.6)		
10–14	19 (6.7)		
15–19	11 (3.9)		
20–29	80 (28.2)		
30–39	40 (14.0)		
40–49	29 (10.2)		
50+	63 (22.2)		
Total	**284 (100.0)**		

*χ* ^2^: chi-square test.

**Table 2:** Frequency of urinary bacterial isolates and their antibiotic resistance patterns from UTI suspected patients attending Gondar University Hospital, Northwest Ethiopia, 2012.

Isolate/*N* (%)	Pattern	Common antibacterial used								
		E *N* (%)	CN *N* (%)	PEN *N* (%)	CAF *N* (%)	TTC *N*(%)	SXT *N* (%)	AMP *N* (%)	CIP *N* (%)	AML *N* (%)
*E. coli* 120 (42.3)	S R	NA	67 (55.8) 53 (44.2)	NA	50 (41.7) 70 (58.3)	25 (20.8) 95 (79.2)	28 (23.3) 92 (76.7)	20 (16.7) 100 (83.3)	64 (53.3) 56 (46.7)	15 (12.5) 105 (87.5)
*Klebsiella *spp. 41 (14.4)	S R	NA	21 (51.2) 20 (48.8)	NA	9 (22) 32 (78)	7 (17.1) 34 (82.9)	7 (17.1) 34 (82.9)	2 (4.9) 39 (95.1)	28 (68.3) 13 (31.7)	1 (2.4) 40 (97.6)
CoNS, 35 (12.3)	S R	20 (57.1) 15 (42.9)	ND	10 (28.6) 25 (71.4)	ND	3 (3.6) 32 (91.4)	9 (25.7) 26 (74.3)	10 (28.6) 25 (71.4)	16 (45.7) 19 (54.3)	10 (28.6) 25 (71.4)
*S. aureus* 26 (9.2)	S R	12 (46.2) 14 (53.8)	ND	9 (34.6) 17 (65.4)	ND	4 (15.4) 22 (84.6)	5 (9.2) 21 (80.8)	8 (30.8) 16 (69.2)	12 (46.2) 14 (53.8)	7 (26.9) 19 (73.1)
*Proteus* spp. 16 (5.6)	S R	NA	11 (68.8) 5 (31.2)	NA	7 (43.8) 9 (56.2)	2 (12.5) 14 (87.5)	4 (25) 12 (75)	3 (18.8) 13 (81.2)	11 (68.8) 5 (31.2)	4 (25) 12 (75)
*Enterobacter* spp. 16 (5.6)	S R	NA	10 (62.5) 6 (37.5)	NA	6 (37.5) 10 (62.5)	4 (25) 12 (75)	3 (18.8) 13 (81.2)	3 (18.8) 13 (81.2)	12 (75) 4 (25)	3 (18.8) 13 (81.2)
*Citrobacter *spp. 10 (3.5)	S R	NA	1 (10) 9 (90)	NA	3 (30) 7 (70)	1 (10) 9 (90)	2 (20) 8 (80)	2 (20) 8 (80)	4 (40) 6 (60)	1 (10) 9 (90)
*Pseudomonas* spp. 5 (1.8)	S R	NA	ND	NA	0 (0) 5 (100)	5 (100) 0 (0)	0 (0) 5 (100)	2 (40) 3 (60)	2 (40) 3 (60)	2 (40) 3 (60)
*Providencia* spp. 5 (1.8)	S R	NA	2 (40) 3 (60)	NA	2 (40) 3 (60)	2 (40) 3 (60)	1 (20) 4 (80)	1 (20) 4 (80)	3 (60) 2 (40)	1 (20) 9 (80)
*Serratia* spp. 2 (0.7)	S R	NA	2 (100) 0 (0)	NA	0 (0) 2 (100)	0 (0) (100)	0 (0) (100)	0 (0) 2 (100)	2 (100) 0 (0)	1 (50) 1 (50)
*Streptococcus* spp. 5 (1.8)	S R	3 (60) 2 (40)	ND	2 (40) 3 (60)	3 (60) 2 (40)	1 (20) 4 (80)	1 (20) 4 (80)	2 (40) 3 (60)	3 (60) 2 (40)	4 (80) 1 (20)
*Salmonella* spp. 1 (0.4)	S R	0 0	1 (100) 0 (0)	0 0	1 (100) 0 (0)	0 (0) 1 (100)	0 (0) 1 (100)	0 (0) 1 (100)	1 (100) 0 (0)	0 (0) 1 (100)

AMP: ampicillin, AML: amoxicillin, PEN: penicillin G, CIP: ciprofloxacin, E: erythromycin, TTC: tetracycline, CAF: chloramphenicol, SXT: co-trimoxazole, CN: gentamicin, CoNS: coagulase negative *Staphylococci*, S: sensitive, R: resistant, NA: not applicable, and ND: not done.

**Table 3:** Multidrug resistance patterns of isolates from UTI-suspected patients at Gondar University Hospital, 2012.

Isolate	*N* (%)	Multidrug resistance, *N* (%)							
		R0	R1	R2	R3	R4	R5	R6	R7
*E. coli *	120 (42.3)	15 (12.5)	3 (2.5)	15 (12.5)	18 (15)	20 (16.7)	18 (15)	21 (17.5)	9 (7.5)
*Klebsiella* spp.	41 (14.4)	0 (0)	4 (9.8)	4 (9.8)	5 (12.2)	12 (29.3)	10 (24.4)	4 (9.8)	2 (4.9)
CoNS	35 (12.3)	1 (2.9)	6 (17.4)	8 (22.9)	5 (14.3)	3 (8.6)	5 (14.3)	5 (14.3)	2 (5.7)
*S. aureus *	26 (9.2)	1 (3.8)	1 (3.8)	3 (11.5)	2 (7.7)	11 (42.3)	3 (11.5)	3 (11.5)	1 (3.8)
*Enterobacter* spp.	16 (5.6)	1 (6.3)	3 (18.8)	2 (12.5)	2 (12.5)	2 (12.5)	4 (25)	2 (12.5)	0 (0)
*Proteus* spp.	16 (5.6)	1 (6.3)	1 (6.3)	2 (12.5)	2 (12.5)	5 (31.3)	3 (18.8)	2 (12.5)	0 (0)
*Streptococcus* spp.	5 (1.5)	0 (0)	3 (30)	0 (0)	3 (30)	2 (20)	1 (10)	1 (10)	0 (0)
*Citrobacter* spp.	10 (3.5)	1 (10)	1 (10)	1 (10)	2 (20)	2 (20)	1 (10)	0 (0)	2 (20)
*Providencia* spp.	5 (1.8)	1 (20)	0 (0)	1 (20)	1 (20)	1 (20)	0 (0)	1 (20)	0 (0)
*Pseudomonas* spp.	5 (1.8)	0 (0)	0 (0)	0 (0)	0 (0)	1 (20)	2 (40)	2 (40)	0 (0)
*Serratia *spp.	2 (0.7)	0 (0)	0 (0)	0 (0)	0 (0)	1 (50)	1 (50)	0 (0)	0 (0)
*Salmonella* spp.	1 (0.4)	0	0		0	2 (50)	1 (100)	0	0

R: resistant to 0, 1, 2, 3, 4, 5, 6, and 7 antibiotics, respectively.

**Table 4:** Comparison of urinary bacterial isolates from UTI suspected patients at Gondar University Hospital in 2002 and 2012.

Isolate	2002∗	2012	*χ* ^2^	*P* value
	*N* (%)	*N* (%)		
*E. coli *	78 (45.3)	120 (42.3)	1.15	0.29
*Klebsiella* spp.	18 (10.5)	41 (14.4)	1.08	0.3
CoNS∗∗	Not isolated	35 (12.3)	0	0
*S.aureus *	31 (18)	26 (9.2)	9.64	0.002
*Enterobacter* spp.	3 (1.7)	16 (5.6)	3.94	<0.001
*Proteus* spp.	7 (4.1)	16 (5.6)	0.4	0.53
*Streptococcus* spp.	4 (2.3)	5 (1.8)	0.4	0.53
*Citrobacter* spp.	10 (5.5)	10 (3.5)	1.6	0.2
*Pseudomonas* spp.	1 (0.6)	5 (1.8)	0	0.42
*Salmonella* spp.	3 (1.7)	1 (0.4)	0	1.00
*Serratia* spp.	2 (1.2)	2 (0.7)	0	0.63
*Hafnia alvei *	1 (0.6)	Not isolated	0	0
Others	Not isolated	7 (2.5)	0	0

Total	172 (41)	284 (52.8)	19.7	<0.001

^*^Moges et al., 2002 (East African Journal) [1]; ∗∗coagulase negative *Staphylococci*.

**Table 5:** Percentage of isolates with antimicrobial resistance in 2002 and 2012.

Antibiotic	*Escherichia coli* (%)		*Klebsiella* spp. (%)		CoNS^#^ (%)		*Staphylococcus aureus* (%)		*Citrobacter *spp. (%)		*Proteus* spp. (%)	
	2002	2012	2002	2012	2002	2012	2002	2012	2002	2012	2002	2012
Erythromycin	NA	NA	NA	NA	71.5∗∗	37.2	19.4	50.0∗	NA	NA	NA	NA
Gentamicin	14.1	34.2∗	33.3	39	21.4∗∗	0	22.6∗∗	0	50	50	28.6	25
Penicillin	NA	NA	NA	NA	57.1	48.6	38.7	50.0	NA	NA	NA	NA
Chloramphenicol	42.3	48.3	77.8	70.7	50	0	51.6∗∗	0	80	50	71.4	56.2
Tetracycline	68	62.5	77.8∗∗	61	71.4	80.8	71	73.1	80	80	100	62.4
Co-trimoxazole	56.4	70	72.2	78	57.1	57.1	48.4	73.1	90	80	71.4	75
Ampicillin	69.2	56.7	94.4	70.7	21.4	48.6	25.8	50	100∗∗	50	71.4	62.4
Ciprofloxacin	0	30∗	0	24.4∗	0	25.7∗	6.5	38.4∗	0	30∗	0	18.8∗
Amoxicillin	NA	37	ND	43.9	ND	28.6	NA	38.5	ND	20	NA	50

Total	78 (45.3)	120 (42.3)	18 (10.5)	41 (14.4)	14 (8.1)	35 (12.3)	31 (18)	26 (9.2)	10 (5.8)	10 (3.5)	7 (4.1)	16 (5.6)

^*^ *P* < 0.05 compared with results for 2002; ∗∗*P* < 0.05 compared with results for 2012; ^#^coagulase negative *Staphylococci*; ND: not done; NA: not applicable.

**Table 6:** Comparison of multidrug resistance patterns of urinary isolates from UTI suspected patients at Gondar University Hospital in 2002 and 2012.

Isolate/study periods	*N* (%)	R2	R3	R4	R5	R6	R7
*E. coli *							
2002	78 (45.3)	5 (6.4)	19 (24.4)	14 (17.9)	11 (14.1)	1 (1.3)	1 (1.3)
2012	120 (42.3)	15 (12.5)	18 (15)	20 (16.7)	18 (15)	21 (15.5)	9 (7.5)
*Klebsiella* spp.							
2002	18 (10.5)	1 (5.6)	1 (5.6)	7 (38.9)	5 (27.8)	0	0
2012	41 (14.4)	4 (9.8)	5 (12.2)	12 (29.3)	10 (24.4)	4 (4.9)	2 (4.9)
*Proteus* spp.							
2002	7 (4.0)	0	0	0	4 (57.1)	1 (14.3)	0
2012	16 (5.6)	2 (12.5)	2 (12.5)	5 (31.3)	3 (18.8)	2 (12.5)	0
*Citrobacter* spp.							
2002	10 (5.8)	1 (10.0)	1 (10.0)	2 (20.0)	5 (50.0)	0	0
2012	10 (3.5)	1 (10)	2 (30)	2 (20)	1 (10)	1 (10)	0
CoNS							
2002	14 (8.1)	2 (14.3)	3 (21.4)	1 (7.1)	0	1 (7.1)	1 (7.1)
2012	35 (12.3)	8 (22.9)	5 (14.3)	3 (8.6)	5 (14.3)	5 (14.3)	5 (5.7)
*S. aureus *							
2002	31 (18.0)	1 (3.2)	3 (9.7)	7 (22.6)	2 (6.5)	2 (6.5)	3 (9.7)
2012	26 (9.2)	3 (11.5)	2 (7.7)	11 (42.3)	3 (11.5)	1 (3.8)	1 (3.8)

^*^Resistant to 2, 3, 4, 5, 6, and 7 antibiotics, respectively.
